# Insecticide resistance to organophosphates in *Culex pipiens* complex from Lebanon

**DOI:** 10.1186/1756-3305-5-132

**Published:** 2012-07-03

**Authors:** Mike A Osta, Zeinab J Rizk, Pierrick Labbé, Mylène Weill, Khouzama Knio

**Affiliations:** 1 Department of Biology, American University of Beirut, Bliss Street, Beirut, 11072020, Lebanon; 2 Team Genomics of Adaptation, CNRS UMR 5554, Institut des Science de l’Evolution, Université Montpellier 2, Place E. Bataillon, 34095, Montpellier cedex 05, France

**Keywords:** Insecticide resistance, Acetylcholine esterase, *Culex pipiens*

## Abstract

**Background:**

Analysis of *Culex pipiens* mosquitoes collected from a single site in Lebanon in 2005, revealed an alarming frequency of *ace-1* alleles conferring resistance to organophosphate insecticides. Following this, in 2006 the majority of municipalities switched to pyrethroids after a long history of organophosphate usage in the country; however, since then no studies have assessed the impact of changing insecticide class on the frequency of resistant *ace-1* alleles in *C. pipiens*.

**Methods:**

*C. pipiens* mosquitoes were captured indoors from 25 villages across the country and subjected to established methods for the analysis of gene amplification at the *Ester* locus and target site mutations in *ace-1* gene that confer resistance to organophosphates.

**Results:**

We conducted the first large-scale screen for resistance to organosphosphates in *C. pipiens* mosquitoes collected from Lebanon. The frequency of carboxylesterase (*Ester*) and *ace-1* alleles conferring resistance to organophosphates were assessed among *C. pipiens* mosquitoes collected from 25 different villages across the country between December 2008 and December 2009. Established enzymatic assay and PCR-based molecular tests, both diagnostic of the major target site mutations in *ace-1* revealed the absence of the F290V mutation among sampled mosquitoes and significant reduction in the frequency of G119S mutation compared to that previously reported for mosquitoes collected from Beirut in 2005. We also identified a new duplicated *ace-1* allele, named *ace-1*^*D13*^, exhibiting a resistant phenotype by associating a susceptible and a resistant copy of *ace-1* in a mosquito line sampled from Beirut in 2005. Fisher’s exact test on *ace-1* frequencies in the new sample sites, showed that some populations exhibited a significant excess of heterozygotes, suggesting that the duplicated allele is still present. Starch gel electrophoresis indicated that resistance at the *Ester* locus was mainly attributed to the *Ester*^*2*^ allele, which exhibits a broad geographical distribution.

**Conclusions:**

Our analysis suggests that the frequency of resistant *ace-1* alleles in mosquito populations can be downshifted, and in certain cases (F290V mutation) even eliminated, by switching to a different class of insecticides, possibly because of the fitness cost associated with these alleles.

## Background

An important global strategy to contain mosquito-borne diseases is vector control using chemical insecticides. However, the strong dependence on insecticides for mosquito control worldwide and the use of such chemicals in agriculture has led to the physiological resistance of important mosquito vectors in recent years, including *Anopheles gambiae*[1,2], *Aedes aegypti*[3-5], and *Culex pipiens*[6,7]. Hence, monitoring insecticide resistance in mosquito populations is crucial in order to ensure the sustainability of vector control programs [8].

Lebanon is a temperate country where two potentially important mosquito vectors of disease are prevalent, *C. pipiens,* which transmits filarial worms, West Nile (WNV) and several encephalitis viruses [9,10], and *Aedes albopictus* the vector for Chikungunya (CHIKV) [11] and dengue viruses (DENV) [12]. *Ae.aegypti*, the primary vector of DENV is not present in the country. Despite the prevalence of potential vectors of disease, arboviral diseases are absent from Lebanon with the exception of some cases of WNV infections [13], while WNV has been responsible for numerous and iterative outbreaks in Israel [14], a country at the southern border of Lebanon. In the past, however, both DENV [15] and WNV [16] were highly prevalent in the country and a Dengue epidemic affected thousands of individuals in Beirut between the years 1945 and 1946 [15].

Mosquito control in Lebanon depended heavily on organophosphate (OPs) usage before the year 2006. The most commonly used OPs included, dichlorvos, malathion, diazinon and chlorpyriphos. However, after that date the use of OPs dwindled; dichlorvos, malathion and diazinon were eventually discontinued, while chlorpyriphos has remained in use in a limited number of villages. On the other hand, there has been a significant shift towards the use of pyrethroids in most villages, according to the feedback obtained from several municipalities and major insecticide distributors across the country. The most commonly used pyrethroids are alpha-cypermethrin, deltamethrin and tetramethrin. In Lebanon, insecticides are used almost exclusively to control adult mosquito populations by spraying along the roads in villages and around houses, while no strategies exist to identify and treat larval habitats. Resistance to OPs can be metabolic or due to target site modifications. The former is characterized by the amplification of esterases A and B that sequester these insecticides [17], preventing them from reaching their target, the *ace-1* gene-encoded acetylcholinesterase. Target-site modifications are due to three distinct mutations in *ace-1*, resulting in three substitutions, G119S found in several mosquito species, F290V found only in *Culex pipiens*, and F331W found only in *Culex tritaeniorhynchus,* (numbered according to *Torpedo californica ace*[18]), which independently render the enzyme less sensitive to OP insecticides.

Data from *C. pipiens* mosquitoes sampled in 2005 from Beirut indicated a high frequency of both G119S and F209V mutations [19]. Here, we conducted a large-scale one-year survey, between December 2008 and December 2009, to measure the impact of switching to pyrethroids on the residual OP resistance in *C. pipiens* mosquito populations. The study involved analysis of gene amplification at the *Ester* locus and target site mutations in *ace-1* gene in mosquitoes captured indoors across the country.

## Methods

### Collection sites and mosquito strains

**Figure 1:**
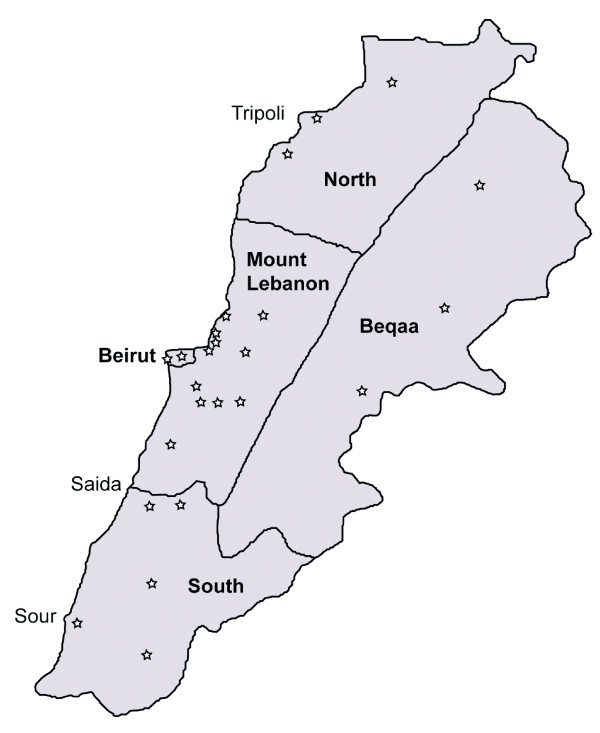
***C. pipiens *collection sites in Lebanon.** In bold are the five geographical regions from where mosquitoes were collected. Stars refer to the collection sites.

*C. pipiens* mosquitoes were collected indoors from 25 villages across Lebanon (Figure 1) in a one year survey between December 2008 and December 2009. *C. pipiens* reference strains included: SLAB, the susceptible strain lacking overproduced esterases [20]; SA2, a resistant strain homozygous for *Ester*^*2*^, characterized by overproduction of esterases A2-B2 [21], and the resistant SR strain homozygous for the G119S mutation [22]. Mosquitoes were reared at room temperature. Larval stages were kept in plastic trays (30 cm x 20 cm) and fed ground fish food (PRODAC), while adult mosquitoes were kept on 10% sucrose solution.

### Starch gel electrophoresis

The frequency of *Ester*^*2*^ (A2-B2) locus among the sampled mosquitoes was determined by Starch gel electrophoresis using TEM 7.4 buffer systems and revealed according to Pasteur *et al.*[23]. The reference strain SA2 [21] was used as a control for esterase amplification at *Ester*^*2*^.

### Detection of the G119S and F290V mutations in *ace-1*

The G119S mutation was detected using a diagnostic PCR test followed by RFLP, as previously described [24]. Briefly, legs of individual mosquitoes were ground in extraction buffer (0.1 M Tris–HCl, pH 8.0, 0.01 M EDTA, 1.4 M NaCl, 2% cetyltrimethyl ammonium bromide), DNA was then extracted with chloroform, precipitated in isopropanol, and resuspended in sterile water. A 374 bp amplicon was amplified from exon 3 of *ace-1* gene using the primers CpEx3dir, 5’-CGACTCGGACCCACTCGT-3’, and CpEx3rev, 5’-GACTTGCGACACGGTACTGCA-3’, and the following PCR conditions: 30 cycles, 95 °C for 5 min, 95 °C for 40 sec, 60 °C for 1 min and 72 °C for 50 sec. Since the G119S mutation in exon 3 creates an AluI restriction site [24], the PCR product was digested with AluI (this generates two fragments of 272 and 102 bp) and products were analyzed on 1.5% agarose gel. Detection of the F290V mutation was performed using the PASA diagnostic test as previously described [25] except that the PCR conditions were modified as follows: 30 cycles, 95 °C for 5 min, 95 °C for 40s, 61 °C for 1 min and 72 °C for 50s.

### Témoin-dichlorvos-propoxur- (TDP) test

The TDP test was performed exactly as described by Alout *et al.*[19] to identify all possible phenotypes at the *ace- 1* locus: phenotype [V], mosquitoes containing only the F290V mutated enzyme; [RR], mosquitoes containing only the G119S mutated enzyme, [SS] those containing susceptible enzyme; [VS], [VR], [RS] and [VRS], mosquitoes containing two or three (VRS) enzyme forms.

### Detection of *ace-1* gene duplications

The genetic test developed by Labbé *et al.*[6] was used to detect the presence of duplicated alleles. Briefly, females from field *C. pipiens* populations were crossed with males from the susceptible reference SLAB strain. After blood feeding, females were isolated individually, allowed to produce egg rafts and then phenotyped using the TDP enzymatic test [25]. The progeny of each female appearing as heterozygotes [R/S] were reared separately and second instar larvae selected with 1 mg/l propoxur, a dose that kills all susceptible [S/S] individuals. Only the progenies of females carrying a duplicated allele displayed no mortality following the exposure to this insecticide. Real heterozygous [R/S] non-duplicated individuals generate [S/S] individuals in their progenies after crossing with susceptible males (for more explanations, see Figure 2 in [6]). DNA was extracted from frozen females, a fragment of the *ace-1* gene encompassing intron 2 and exon 3 was PCR-amplified, and the products were cloned (to separate the different copies), as previously described [6]. Sequences were then analyzed using the Mega 5 software (http://www.megasoftware.net/[26]): Beirut duplicated allele sequences were aligned with known duplicated alleles using ClustalW and a similarity tree was built using Neighbor-Joining with default parameters.

**Figure 2:**
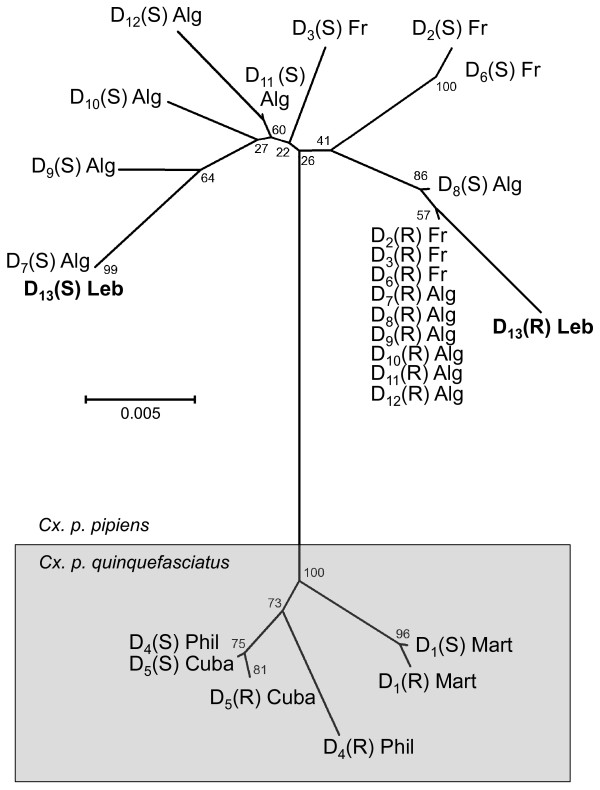
**Genetic distance tree of *ace-1 *sequences from *C. pipiens *carrying a duplicated haplotype.** The similarity of the various duplicated *ace-1* alleles was computed using the Neighbor-Joining method and the genetic distance tree is shown. Sequences of intron 2 and exon 3 were used and positions containing gaps and missing data were eliminated. There were a total of 502 positions in the final dataset. These analyses were conducted in MEGA5 [26]. D_13_(S) and D_13_(R) copies of the Lebanon duplicated allele are in bold characters. Geographical origins of each duplicated allele are indicated (Fr: France; Alg: Algeria; Phil: Philippines, Mart: Martinique).

Due to the similarity of duplicated allele sequences with non-duplicated *ace-1* alleles, it is not possible to directly detect the presence of the duplicated allele in natural populations [6,27]. However, an excess of heterozygote phenotypes at the *ace-1* locus have been shown to suggest robustly the presence of a duplicated allele in a population [28,29]. Thus, excess of heterozygotes (as indicated by a negative F_is_, i.e. a significant departure from the expected frequencies under Hardy-Weinberg assumptions) was tested for each sampled population using the Genepop software (Fisher's exact tests, [30]).

## Results and discussion

### Prevalence of *Ester*^*2*^ in sampled mosquitoes

There are many *Ester* alleles associated with resistance to OPs, the most common type is *Ester*^*2*^, which exhibits a worldwide distribution [31,32]. We used starch gel electrophoresis to identify amplifications at the *Ester* locus. The reference strain SA2 [21] was used as a control for the presence of the *Ester*^*2*^ allele, since preliminary data indicated a high frequency of this allele in a sample collected from Beirut in 2005 (M. Weill, unpublished data). The 2008–2009 survey indicated that approximately 40.8% of sampled mosquitoes carried the *Ester*^*2*^ (A2-B2) resistance allele (Table 1), while the rest showed no other resistance allele at that locus. This, apparently high prevalence, probably reflects the continuous, though limited use of chlorpyriphos in some villages. Moreover, cypermethrin, a pyrethroid used in many municipalities in Lebanon, is known in other organisms to select for resistance due to elevated esterase production [33].

**Table 1:** **Frequency of*Ester***^***2***^**phenotype in the mosquito populations sampled**

**Location**	**N**	**None**	**[*Ester***^***2***^**]**
Beirut	64	0.67 (43)	0.33 (21)
Mount Lebanon	90	0.60 (54)	0.40 (36)
South Lebanon	183	0.51 (93)	0.49 (90)
North Lebanon	153	0.61 (94)	0.39 (59)
Beqaa	134	0.63 (85)	0.37 (49)
**Total number**	624	369	255

Indicated are the frequencies of the normal (None) and *Ester*^*2*^ phenotypes together with the corresponding number of individuals (in brackets) from the different geographical locations. N is the number of mosquitoes analyzed.

### Geographical distribution of AChE1 G119S substitution and evidence for *ace-1* duplication

The analysis of insecticide resistance in a small sample of *C. pipiens* mosquitoes collected from Beirut in 2005 revealed an alarming frequency of the G119S resistant AChE1 distributed as follows [19]: 41.4% [RS], 37.9% [RR], 8.6% [VS] and 12.1% [VRS]. None of the mosquitoes sampled exhibited the [SS] phenotype (susceptible enzyme), suggesting that in 2005 there was indeed a strong selective pressure on the *ace-1* locus due to the past heavy usage of OPs. Here, we screened a mosquito line, that was selected with OP for several generations from this 2005 Beirut sample, for the presence of a duplicated *ace-1* allele as previously reported [6]. The duplicated *ace-1* allele (or haplotype) originated from duplication of the *ace-1* gene (termed *ace-1*^*D*^ or D) which associates a resistant and a susceptible copy (termed D(R) and D(S), respectively), resulting in "permanent heterozygotes" [6]. This screen revealed the presence of a new duplicated allele associating the D_7_S susceptible copy already found in Algeria [34] and a new DR resistant copy (Figure 2), hence exhibiting an overall resistant phenotype. This new allele (as of the complete haplotype) should thereafter be named *ace-1*^*D13*^, according to previous nomenclature [34] (*ace-1*^*D13*^ susceptible and resistant copies are available in GenBank under references JX007790 and JX007791, respectively. All other duplicated alleles can be found in GenBank under references JX007766 to JX007789). Also, considering only the G119S mutation, the *ace-1* allele frequencies in the 2005 sample reported above [19] show a significant departure from the Hardy-Weinberg equilibrium (Genepop, [30], Fisher’s exact-test for an excess of heterozygotes; F_is_ = −0.34, *p*-value =0.018). This provides additional support to the presence of at least one *ace-1* duplicated allele in the 2005 Beirut sample, as previously reported in other locations [27,35].

In our more recent samples collected between December 2008 and December 2009, we identified only two genotypes associated with the *ace-1* locus using PCR analysis; mosquitoes carrying only the susceptible *ace-1* allele [SS] and mosquitoes carrying one susceptible and one resistant *ace-1* alleles [RS]. The frequency of [SS] ranged from 0.74 to 0.82 across the five geographical regions, while [RS] ranged between 0.17 and 0.25 (Table 2). The TDP assay gave similar results: the frequency of [SS] and [RS] were 0.76 and 0.24, respectively, in the pool of sampled mosquitoes. Our data suggest that switching to pyrethroids resulted in a dramatic reduction in frequency of resistant mosquitoes, possibly because of the fitness cost associated with this mutation in the absence of a selecting insecticide [36,37]; Indeed, [RR] mosquitoes, supposedly the ones with the highest fitness cost, disappeared. The G119S mutation is known to significantly reduce the enzymatic activity of AChE1 in cholinergic synapses affecting the behavior of mosquitoes, which is probably the cause of this fitness cost [38]. We did not perform genetic crosses to determine whether the [RS] phenotype represented a heterozygous state or was due to the presence of the duplicated allele, since no mosquito lines were maintained from the 2008–2009 collection sites. However, statistical analyses on the *ace-1* frequencies showed that all populations exhibited an excess of heterozygotes, which was significant in the Mount and South Lebanon populations (Table 2), suggesting that the duplicated allele is still present [27,35]. This type of duplicated allele is thought to be less costly than the single R allele, although it is not always the case [6,35]. However, the observed reduction of [RS] indicates that the duplicated allele is undoubtedly less fit than the susceptible one in the absence of insecticides. We endeavored to collect information on the history of insecticide usage from the municipalities corresponding to the different collection sites; this task was difficult because Lebanon is a free market and there are no unified guidelines for insecticide usage in the country. The information gathered from 31 villages across the country revealed that 5-10% of municipalities are still using chlorpyrifos (an OP) either alone or in combination with pyrethroids, which probably explains why the G119S mutation is still present at low frequencies in *C. pipiens* populations.

**Table 2:** **Frequency of*ace-1*alleles based on diagnostic PCR analysis**

**Location**	**N**	**[SS]**	**[RS]**	**[RR]**	**[V]**	**F**_**is**_	***p*-value**
Beirut	76	0.763 (58)	0.236 (18)	0	0	−0.13	0.32
Mount Lebanon	182	0.73 (133)	0.27 (49)	0	0	−0.15	**0.02**
South Lebanon	159	0.742 (118)	0.258 (41)	0	0	−0.14	**0.05**
North Lebanon	120	0.80 (96)	0.20 (24)	0	0	−0.11	0.28
Beqaa	107	0.822 (88)	0.178 (19)	0	0	−0.09	0.41
**Total number**	644	493	151	0	0	-	-

Indicated are the frequencies for each genotype together with the corresponding number of individuals in brackets. N is the number of mosquitoes analyzed. SS, susceptible mosquitoes; RS, mosquitoes heterozygous for G119S; RR, mosquitoes homozygous for G119S; V, mosquitoes containing the F290V mutation. The F_is_ value indicates whether there is an excess (negative value) or a deficit (positive value) of heterozygotes. The *p*-value (Genepop [30], Fisher’s exact test) is also indicated. Significant values are in bold.

Interestingly, the F290V substitution that was previously detected at low frequency only in five Mediterranean countries including Lebanon [19], was not detected in the present study using both the PASA (Table 2) and TDP tests, despite the large samples collected across the country. A plausible explanation for the disappearance of the F290V substitution from natural populations is the increased resort to pyrethroids, which do not select for this mutation. Moreover, even in municipalities where chlorpyriphos is still used, the resistance conferred by the F290V mutation to this insecticide is 150-fold weaker than that conferred by G119S, according to Alout *et al.*[25]. Thus, high fitness cost in addition to weak insecticide selection of the F290V AChE1 may explain the loss of this mutation from *C. pipiens* in Lebanon. The reason why F290V was prevalent at low frequencies in samples collected from Beirut in 2005 may be due to the insecticide dichlorvos which was commonly used at that time together with chlorpyriphos. F290V was shown to confer approximately 10 fold higher resistance to dichlorvos compared to the G119S mutation [25].

## Conclusions

In summary, this study shows that the frequencies of resistant *ace-1* alleles carrying the G119S and F290V substitutions were dramatically reduced in *C. pipiens* mosquitoes collected between 2008 and 2009 when compared to those recorded in 2005. This is probably due to increased dependence on pyrethroid insecticides in recent years that do not select for these mutations. We propose that using a rotation system, whereby the use of different classes of insecticides is alternated on a yearly basis, should maintain resistance *ace-1* alleles at a low frequency in *C. pipiens* mosquitoes. Nevertheless, cross-resistance between pyrethroids and OP through elevated esterases should be considered and evaluated.

## Competing interests

The authors declare that they have no competing interests.

## Authors’ contributions

MAO, KK and MW conceived the study. ZR performed the diagnostic PCR analysis and TDP enzymatic assays. Statistical analysis and *ace-1* duplication studies were performed by MW and PL. All authors were involved in data analysis and interpretation. MAO drafted the manuscript. KK, PL and MW critically revised the manuscript. All authors read and approved the final version.
